# Supplementary material for: The phosphatase PTEN links platelets with immune regulatory functions of mouse T follicular helper cells
Source: Nat Commun. 2022 May 19;13:2762. doi: 10.1038/s41467-022-30444-y (PMC9120038; doi:10.1038/s41467-022-30444-y)

The phosphatase PTEN links platelets with immune regulatory functions of T follicular helper cells

Inventory of Supporting Information

1. **Supplementary Figures and figure legends**

   Supplementary Figure 1 *Pten*$^{fl/fl}$*Pf4-Cre* mice display normal B cell development in bone marrow.
   Supplementary Figure 2 Expansion of IFNg^{+}, IL-4^{+}, IL17^{+} T subsets in pLNs from *Pten*$^{fl/fl}$*Pf4-Cre* mice.
   Supplementary Figure 3 Altered T cell differentiation and excessive Tfh cell response in the spleens of *Pten*$^{fl/fl}$*Pf4-Cre* mice.
   Supplementary Figure 4 *Pten*$^{fl/fl}$*Pf4-Cre* mice displayed normal homeostasis of splenic DCs.
   Supplementary Figure 5 The frequencies of GFP-positive cells in the indicated lineages.
   Supplementary Figure 6 Nonspecific PTEN deficiency in T/B subsets has a very limited effect on indicated cell populations.
   Supplementary Figure 7 The expansion of activate, Th1, Tfh and GC-B subsets is cell-extrinsic.
   Supplementary Figure 8 The production and secretion of cytokines in *Pten*$^{fl/fl}$ and *Pten*$^{fl/fl}$*Pf4-Cre* platelets.
   Supplementary Figure 9-11 Gating Strategy used for flow cytometric analysis in the manuscript figures.
2. **Supplementary Table 1**
3. **Source data for the blot in Supplementary Figure 8b**

Supplementary Figures and figure legends

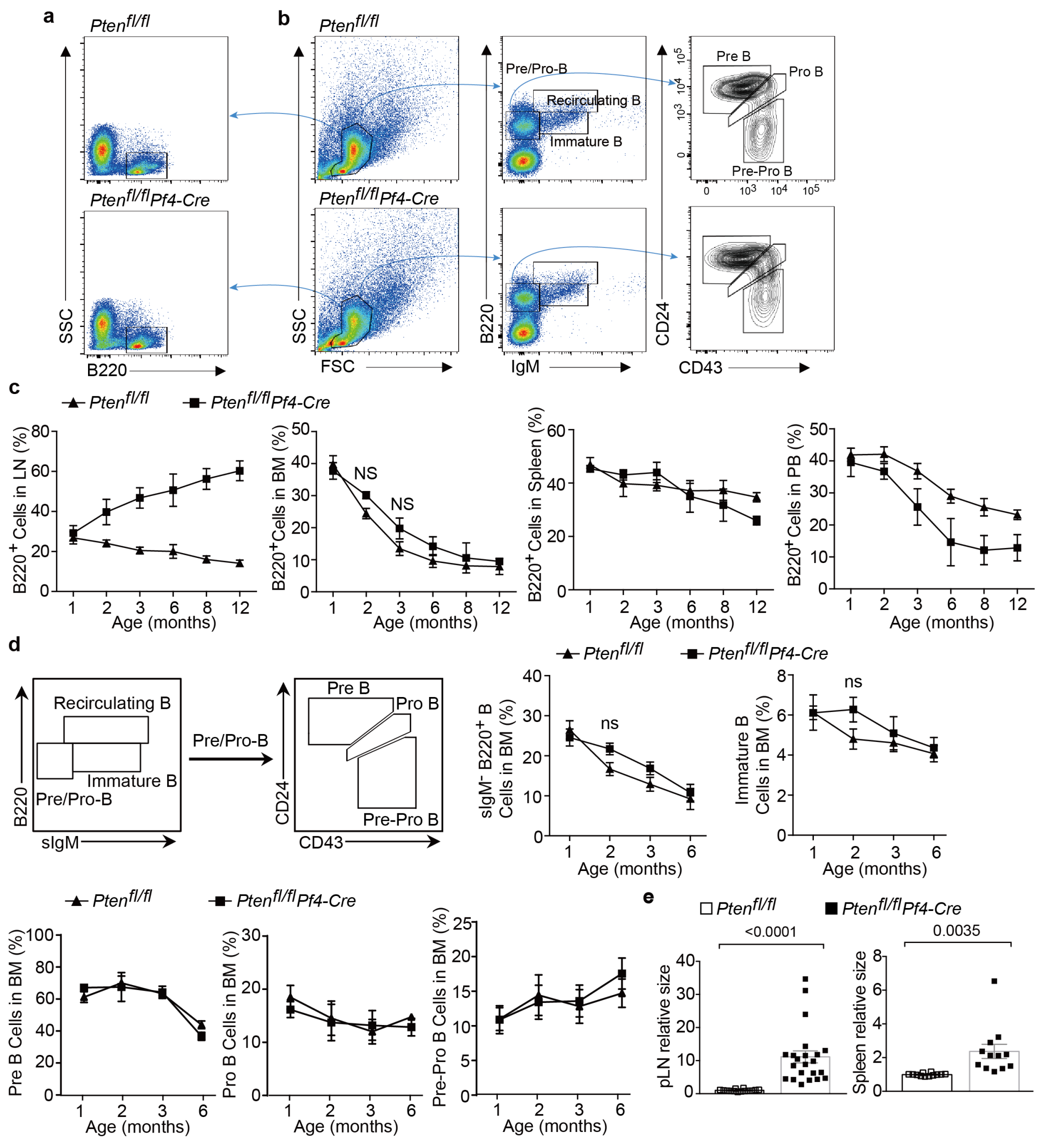

Supplementary Figure. 1

Supplementary Figure 1 *Pten*$^{fl/fl}$*Pf4-Cre* mice display normal B cell development in bone marrow. The mice of indicated ages were subjected for analysis (**a-d**). **a** The gating panels correspond to FACS data panels in **c**. **b** The gating panels correspond to FACS data panels in **d**. **c** Frequencies of B220^{+} B cells in pLNs, bone marrow (BM), spleen (SP) and peripheral blood (PB) from *Pten*$^{fl/fl}$ or *Pten*$^{fl/fl}$*Pf4-Cre* mice at the indicated ages. **d** Frequencies of Pre/Pro B (sIgM^{-}B220^{+}), immature B (sIgM^{+}B220^{+}), recirculating B cells (sIgM^{+}B220^{hi}) and committed B-lineage progenitors in bone marrow, including sIgM^{-}B220^{+}CD24^{+}CD43^{-} Pre B cells, sIgM^{-}B220^{+}CD24^{+}CD43^{+} Pro B cells and sIgM^{-}B220^{+}CD24^{-}CD43^{+} Pre-Pro B cells from mice at the indicated ages. **e** Statistical analysis of the relative size of pLNs and spleens from *Pten*$^{fl/fl}$ (n=14) or *Pten*$^{fl/fl}$*Pf4-Cre* mice (n=22), age and sex matched 6-month-old mice were used. Data are representative of three independent experiments and obtained from 3-6 mice for each genotype. Data (**c-e**) are presented as mean ± s.e.m. ns, not significant. (two-tailed t-test). Source data are provided as a Source Data file.

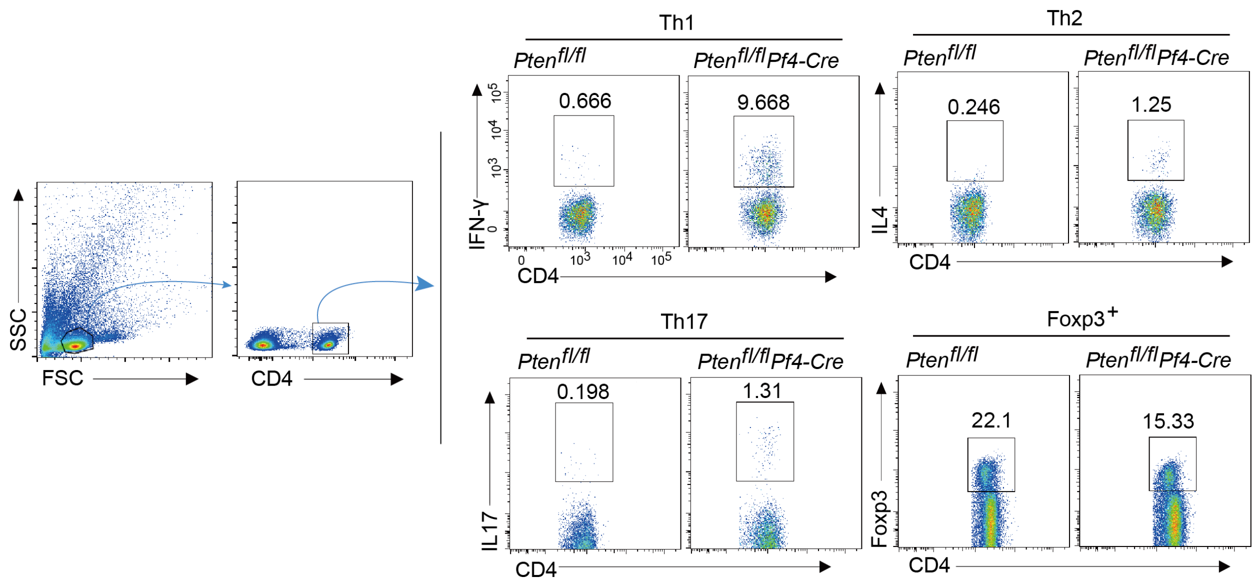

Supplementary Figure. 2

Supplementary Figure 2 Expansion of IFNg$^+$, IL-4$^+$, IL17$^+$ T subsets in pLNs from *Pten*$^{fl/fl}$*Pf4-Cre* mice. The pLNs of 6-month-old mice were subjected for analysis. Representative flow cytometry plots of the proportions of IFNg$^+$, IL-4$^+$, IL17$^+$ and Foxp3$^+$ cells among CD4$^+$ cells in the pLNs from *Pten*$^{fl/fl}$ and *Pten*$^{fl/fl}$*Pf4-Cre* mice, respectively. Results are representative of three independent experiments.

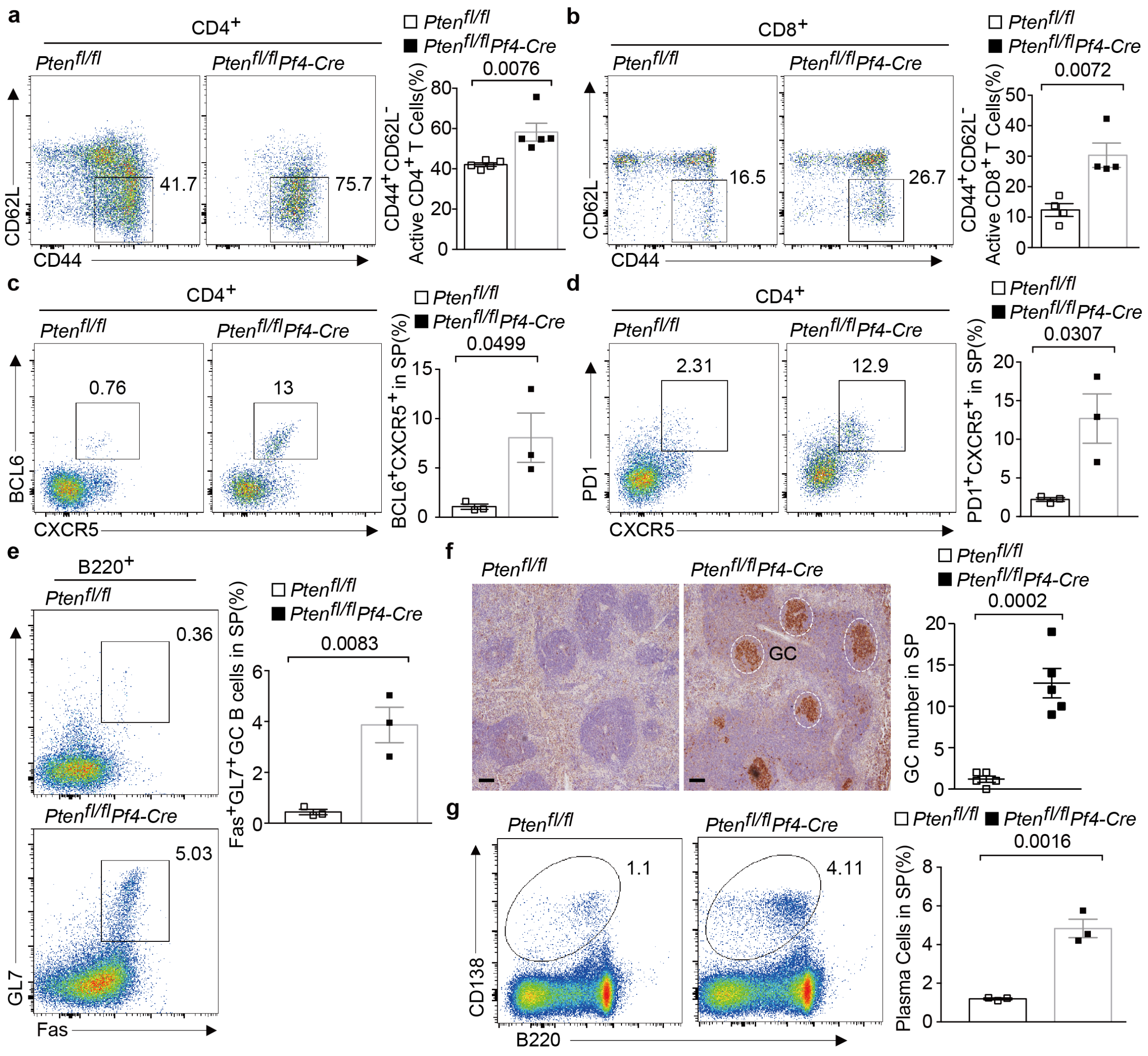

Supplementary Figure. 3

Supplementary Figure 3 Altered T cell differentiation and excessive Tfh cell response in the spleens of *Pten*$^{fl/fl}$*Pf4-Cre* mice. The spleens from around 6-month-old *Pten*$^{fl/fl}$ and *Pten*$^{fl/fl}$*Pf4-Cre* mice were subjected for analysis (**a-g**). **a, b** Flow cytometry analysis and quantification of CD62L and CD44 expression on CD4^{+} (**a**, n=5/group) or CD8^{+} T cells (**b**, n=4/group). **c, d** Flow cytometry analysis and quantification of CXCR5^{+}BCL6^{+} Tfh (**c,** n=3/group) or CXCR5^{+}PD1^{+}Tfh (**d,** n=3/group) cells (gated on live CD4^{+} cells). **e** Flow cytometry analysis and quantifications of Fas^{+}GL7^{+} GC B cells (gated on live B220^{+} cells, n=3/group). **f** Representative PNA staining of splenic sections (Left, scale bars, 100 μm). Germinal centers are identified as PNA-positive clusters and circled with white line. Quantifications of the number of GCs per spleen section are shown at the right (n=3 biologically independent samples). **g** Flow cytometry analysis and quantification of CD138^{+} plasma cells (n=3/group). Data are representative of three independent experiments. Each symbol represents one mouse (**a-g**), and data are presented as mean ± s.e.m. ns, not significance. (two-tailed t-test). Source data are provided as a Source Data file.

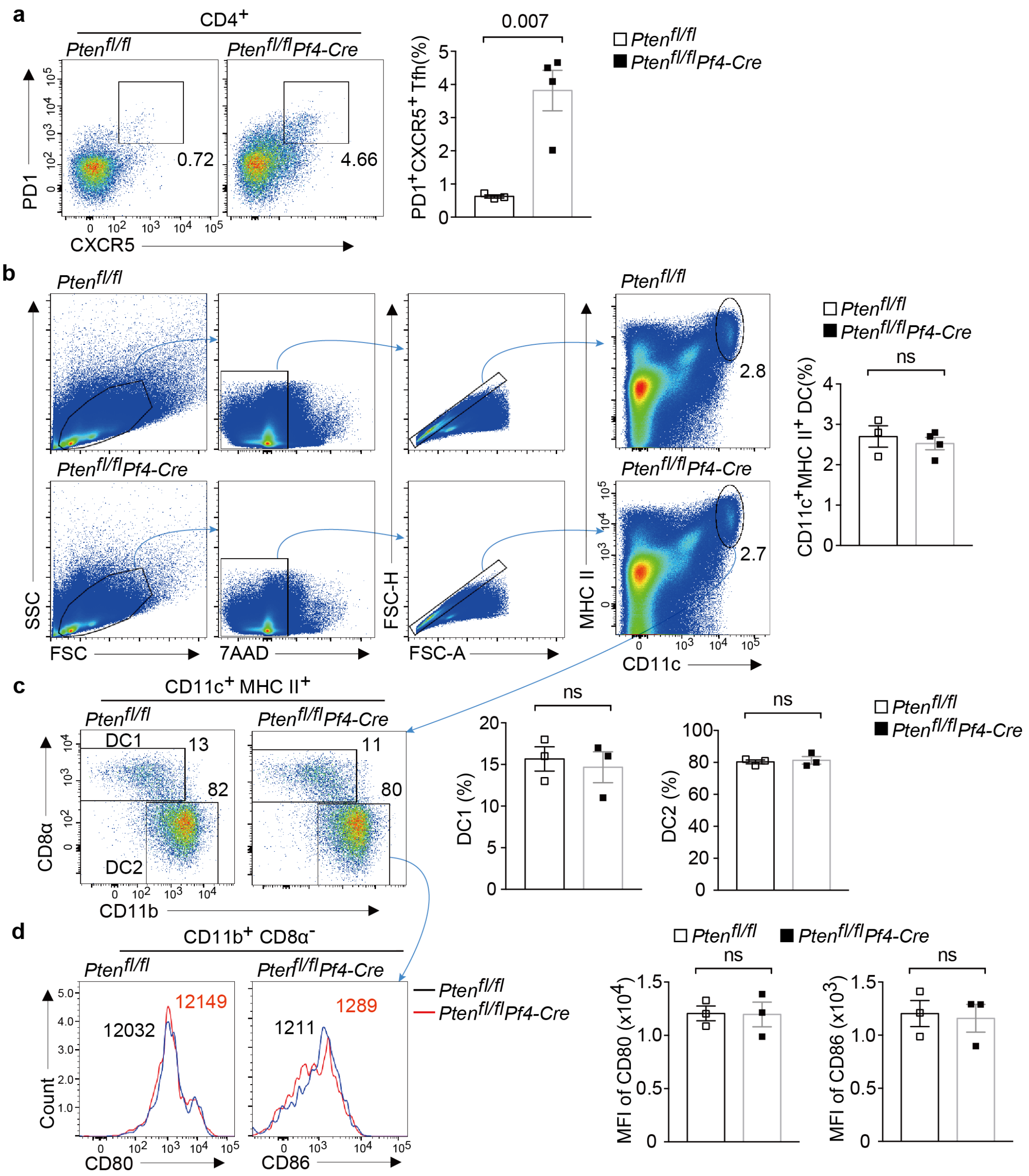

Supplementary Figure. 4

Supplementary Figure 4 ***Pten*$^{\text{fl/fl}}$*Pf4-Cre* mice displayed normal homeostasis of splenic DCs**. The spleens of 3- or 5-month-old mice were subjected for analysis (**a-d**). **a** Flow cytometry plots and quantifications of CXCR5^{+} PD1^{+} Tfh cells (*Pten*$^{\text{fl/fl}}$ n=3; *Pten*$^{\text{fl/fl}}$*Pf4-Cre* n=4). **b** Flow cytometry analysis and frequencies of CD11c^{+} MHC II^{+} DCs (*Pten*$^{\text{fl/fl}}$ n=3; *Pten*$^{\text{fl/fl}}$*Pf4-Cre* n=4). **c** Flow cytometry analysis and proportions of cDC1 (CD8a^{+}CD11b^{-}) and cDC2 (CD8a^{-}CD11b^{+}) among DCs (n=3 mice/group). **d** Flow cytometry analysis and quantifications of CD80 and CD86 expression among cDC2 (n=3 mice/group). Each symbol represents one mouse (**a-d**), and data are presented as mean ± s.e.m. ns, not significance. (two-tailed t-test). Results are representative of three independent experiments. Source data are provided as a Source Data file.

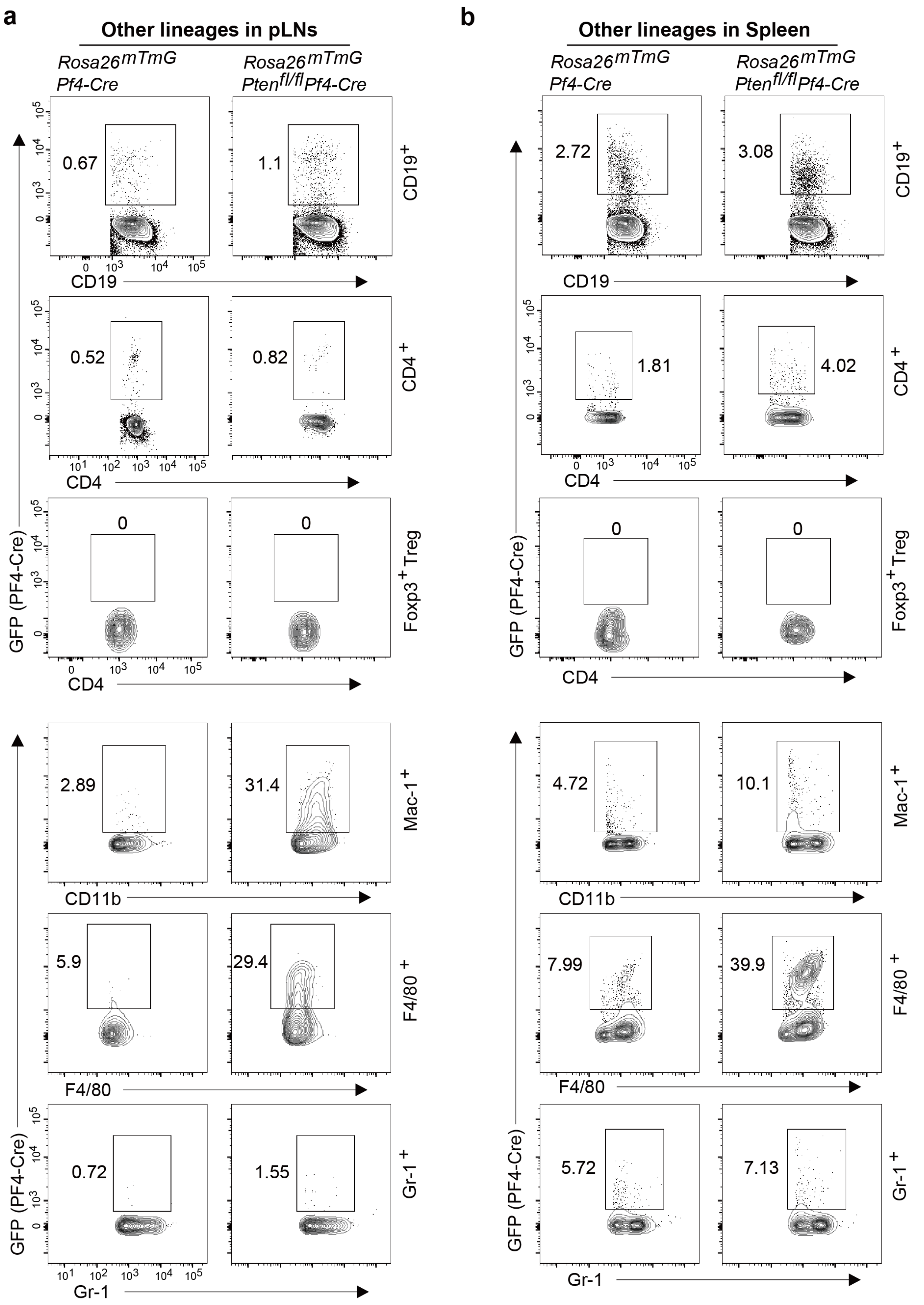

Supplementary Figure. 5

Supplementary Figure 5 The frequencies of GFP-positive cells in the indicated lineages. The pLNs or spleens of 6-month-old mice were subjected for analysis (**a-b**). **a-b** Representative flow cytometry plots of GFP^+ cells among $CD19^+$, $CD4^+$, $Foxp3^+$, Mac-1^+, F4/80^+ and Gr-1^+ cells in the pLNs (**a**) or spleens (**b**) of *Rosa26*$^{mT/mG}$*Pf4-Cre* and *Rosa26*$^{mT/mG}$*Pten*$^{fl/fl}$*Pf4-Cre* mice, respectively. Results are representative of three independent experiments.

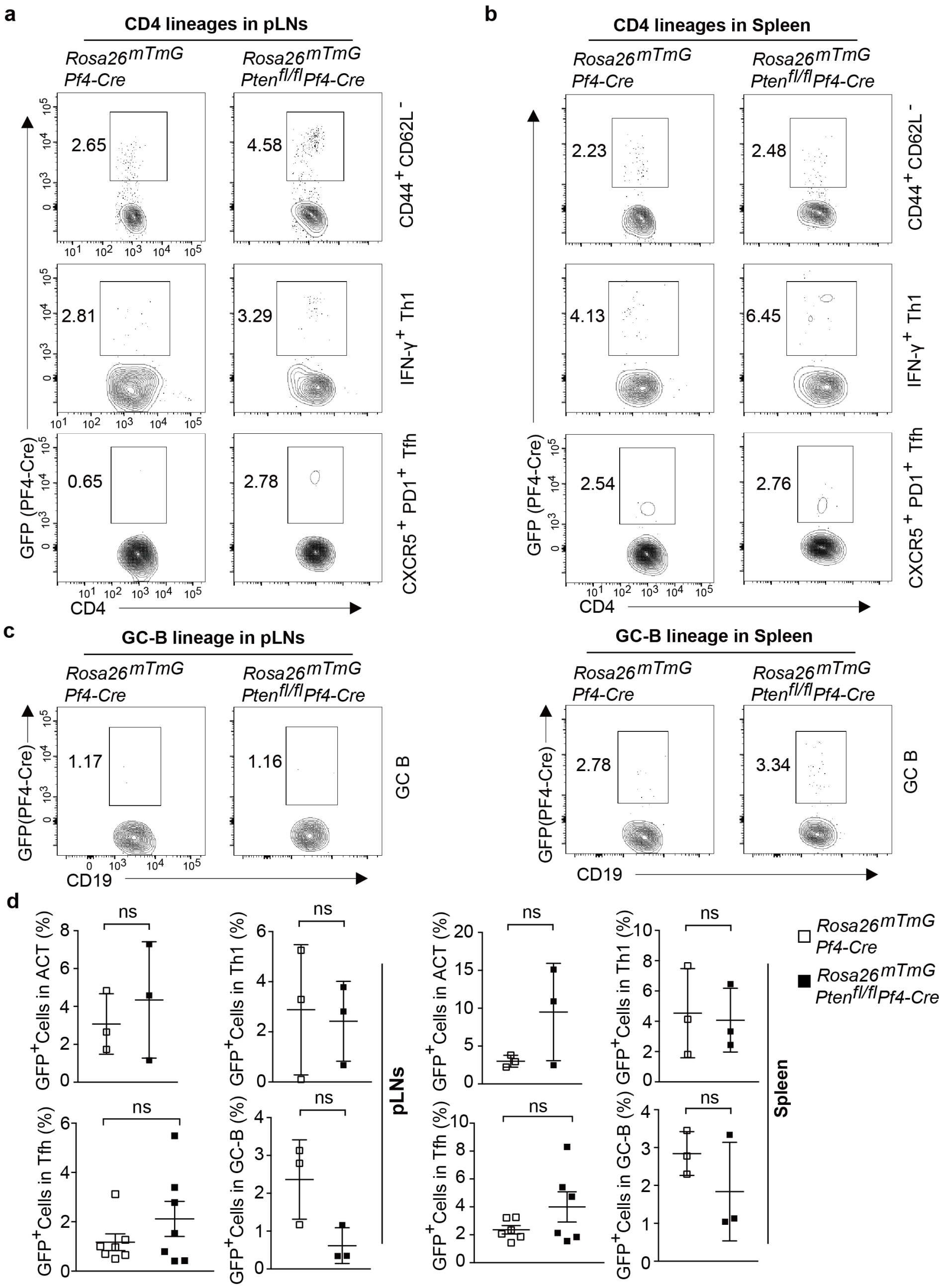

Supplementary Figure. 6

Supplementary Figure 6 Nonspecific PTEN deficiency in T/B subsets has a very limited effect on indicated cell populations. The pLNs or spleens of 6-month-old mice were subjected for analysis (**a-d**). **a-c** Representative flow cytometry plots of GFP^{+} cells among CD44^{+}CD62L^{-} active T cell (ACT), IFN-γ^{+} Th1, and CXCR5^{+}PD1^{+} Tfh (**a, b**) and GC-B cells (**c**) in the pLNs or spleens of *Rosa26*$^{\mathrm{mT/mG}}$*Pf4-Cre* and *Rosa26*$^{\mathrm{mT/mG}}$*Pten*$^{\mathrm{fl/fl}}$*Pf4-Cre* mice, respectively. **d** Statistical analysis of GFP^{+} cells among indicated cell lineages in pLNs (ACT, n=3; Th1, n=3; Tfh, n=7; GC B, n=3) or spleens (ACT, n=3; Th1, n=3; Tfh, n=7; GC B, n=3) from *Rosa26*$^{\mathrm{mT/mG}}$*Pf4-Cre* and *Rosa26*$^{\mathrm{mT/mG}}$*Pten*$^{\mathrm{fl/fl}}$*Pf4-Cre* mice. Results are representative of three independent experiments and each symbol represents one mouse, and data (**d**) are presented as mean ± s.e.m. ns, not significance. (two-tailed t-test). Source data are provided as a Source Data file.

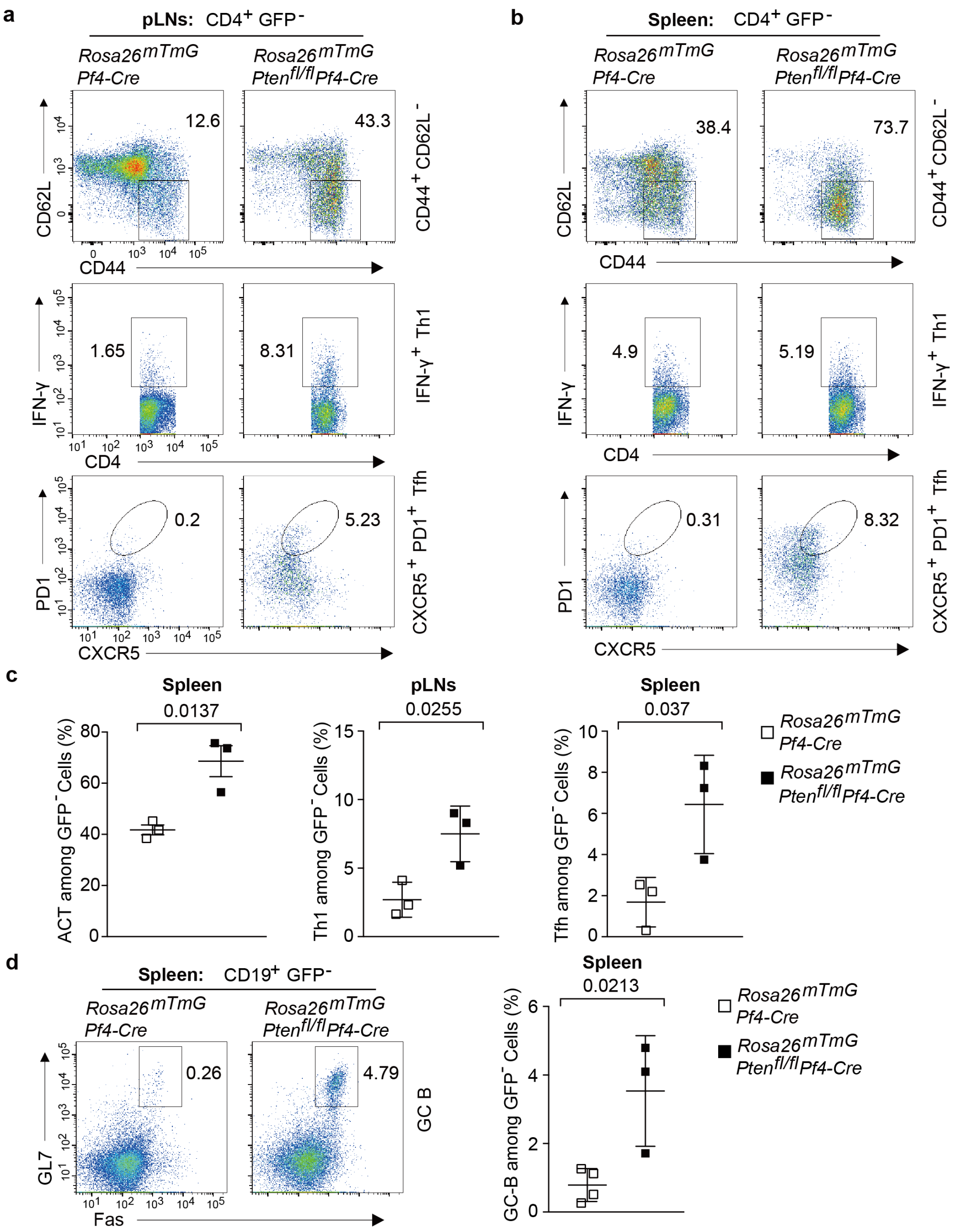

Supplementary Figure. 7

Supplementary Figure 7 The expansion of activate, Th1, Tfh and GC-B subsets is cell-extrinsic. Around 6-month-old *Pten*$^{fl/fl}$ and *Pten*$^{fl/fl}$*Pf4-Cre* mice were subjected for analysis (**a-d**). **a-b** Representative flow cytometry plots of CD44^{+}CD62L^{-} active T cells (ACT), IFN-γ^{+} Th1, CXCR5^{+}PD1^{+} Tfh cells among CD4^{+}GFP^{-} cells in the pLNs (**a**) and spleens (**b**) of indicated mice. **c** Quantifications of the percentages of ACT, Th1 and Tfh among CD4^{+}GFP^{-} cells in pLNs or spleens of *Rosa26*$^{mT/mG}$*Pf4-Cre* (n=3) and *Rosa26*$^{mT/mG}$*Pten*$^{fl/fl}$*Pf4-Cre* (n=3) mice, respectively. **d** Proportions of GL7^{+}Fas^{+} GC-B cells among B220^{+} GFP^{-} subsets in spleen of indicated mice (white square, n=4; black square, n=3). Data are representative of three independent experiments and each symbol represents one mouse, and data (**c, d**) are presented as mean ± s.e.m. (two-tailed t-test). Source data are provided as a Source Data file.

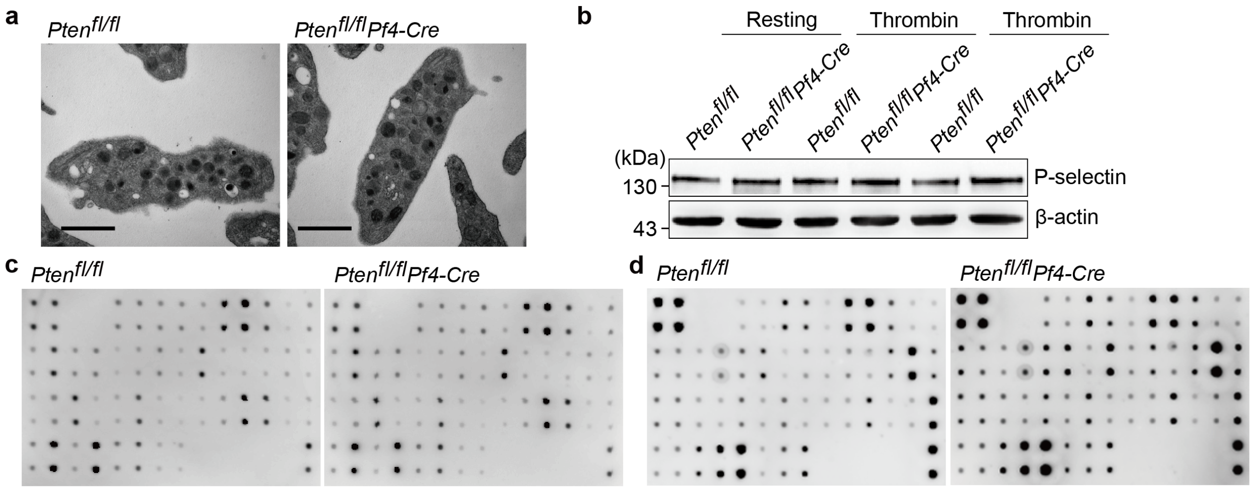

e

	a	b	c	d	e	f	g	h	i	j	k	l	m	n
1	POS	POS	NEG	NEG	bFGF	VEGF-A	TGF-beta 1	EGF	HGF	IGFBP-3	IGF-I	IGF-II	TNF-alpha	DKK-1
2	POS	POS	NEG	NEG	bFGF	VEGF-A	TGF-beta 1	EGF	HGF	IGFBP-3	IGF-I	IGF-II	TNF-alpha	DKK-2
3	IL-1alpha	IL-1beta	IL-1ra	IL-2	IL-3	IL-4	IL-5	IL-6	IL-7	IL-9	IL-10	IL-11	IL-12p40	IL-12p70
4	IL-1alpha	IL-1beta	IL-1ra	IL-2	IL-3	IL-4	IL-5	IL-6	IL-7	IL-9	IL-10	IL-11	IL-12p40	IL-12p70
5	IL-13	IL-15	IL-17	IL-17B	IL-17E	IL-17F	IL-20	IL-21	IL-22	IL-23p19	IL-28/IFN-λ	IL-33	IFN-gamma	LIX
6	IL-13	1L-15	IL-17	IL-17B	IL-17E	IL-17F	IL-20	IL-21	IL-22	IL-23p19	IL-28/IFN-λ	IL-33	IFN-gamma	LIX
7	MCP-1/CCL2	MIP-1alpha	MMP-2	OPG	PF-4	SDF-1alpha	TARC	RANTES	BLANK	BLANK	BLANK	BLANK	BLANK	POS
8	MCP-1/CCL2	MIP-1alpha	MMP-2	OPG	PF-4	SDF-1alpha	TARC	RANTES	BLANK	BLANK	BLANK	BLANK	BLANK	POS

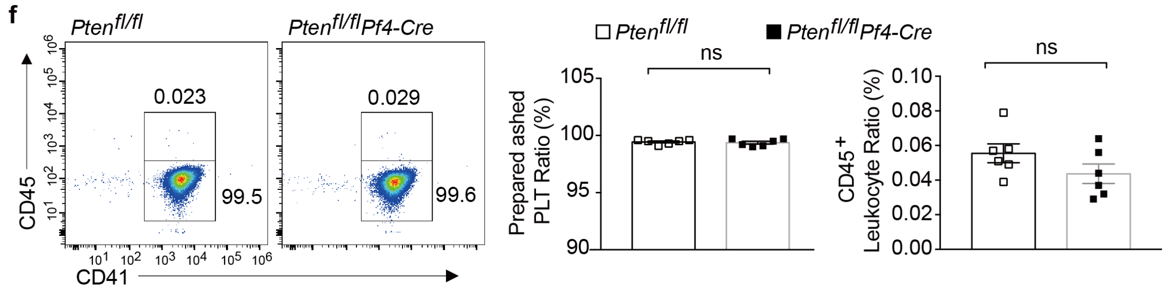

Supplementary Figure. 8

Supplementary Figure 8 The production and secretion of cytokines in *Pten*$^{fl/fl}$ and *Pten*$^{fl/fl}$*Pf4-Cre* platelets. Around 6-month-old *Pten*$^{fl/fl}$ and *Pten*$^{fl/fl}$*Pf4-Cre* mice were subjected for analysis (**a-f**). **a** Representative transmission electron microscopy images of *Pten*$^{fl/fl}$ or *Pten*$^{fl/fl}$ *Pf4-Cre* platelets. Scale bars, 1μm. **b** Western blotting of P-selectin expression in *Pten*$^{fl/fl}$ or *Pten*$^{fl/fl}$*Pf4-Cre* platelets treated with indicated concentrations of Thrombin. **c** Representative images of cytokine arrays of untreated-*Pten*$^{fl/fl}$ or *Pten*$^{fl/fl}$*Pf4-Cre* platelets. **d** Representative images of cytokine arrays of *Pten*$^{fl/fl}$ or *Pten*$^{fl/fl}$*Pf4-Cre* platelets stimulated by 0.1 U/ml Thrombin. **e** The cytokines listed in the array. **f** The purity of prepared platelets, assessed by flow cytometry (n=6 per group in each panels). Data are representative of at least three independent experiments, and data (**f**) are presented as mean ± s.e.m. ns, not significance. (two-tailed t-test). Source data are provided as a Source Data file.

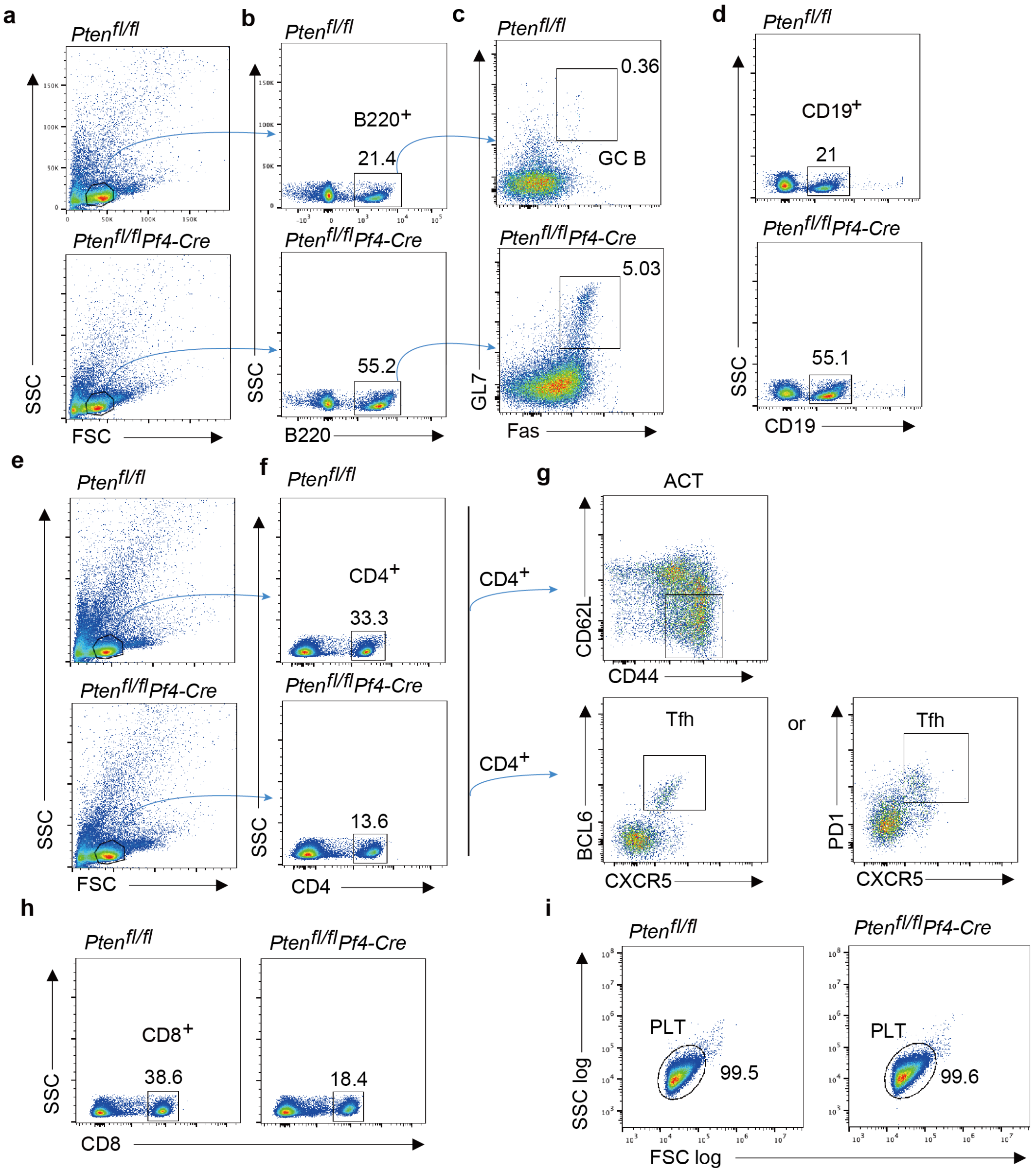

Supplementary Figure. 9

Supplementary Figure 9 Gating Strategy used for flow cytometric analysis of GC B cells, ACT and Tfh cells in the figures. All cell populations were gated on live singlets. **a-c** Gating strategies used for flow cytometric analysis of GC B cells correspond to FACS data panels in figure 2e, figure 5f, Supplementary figure 3e. **a** The gating panels correspond to FACS data panels in the manuscript figure 1c, figure 2g, figure 5c, Supplementary figure 3g. **a, b** The B220^{+} gating panels correspond to FACS data panels in the manuscript figure 1d left panel. **a, d** The CD19^{+} gating panels correspond to FACS data panels in Supplementary figure 5 first row. **e-g** Gating strategies used for flow cytometric analysis of ACT or Tfh cells which correspond to FACS data panels in figure 2a, d, figure 5e and Supplementary figure 3a, c, d, Supplementary figure 4a. **e, f** The CD4^{+} gating panels correspond to FACS data panels in the figure 1d right panel, figure 2c, figure 5a, b d, Supplementary figure 4a, Supplementary figure 5 second row. **e, h** The CD8^{+} gating panels correspond to FACS data panels in the figure 2b, Supplementary figure 3b. **i** The gating panels correspond to FACS data panels in the figure 4b, 4g, and Supplementary figure 8f.

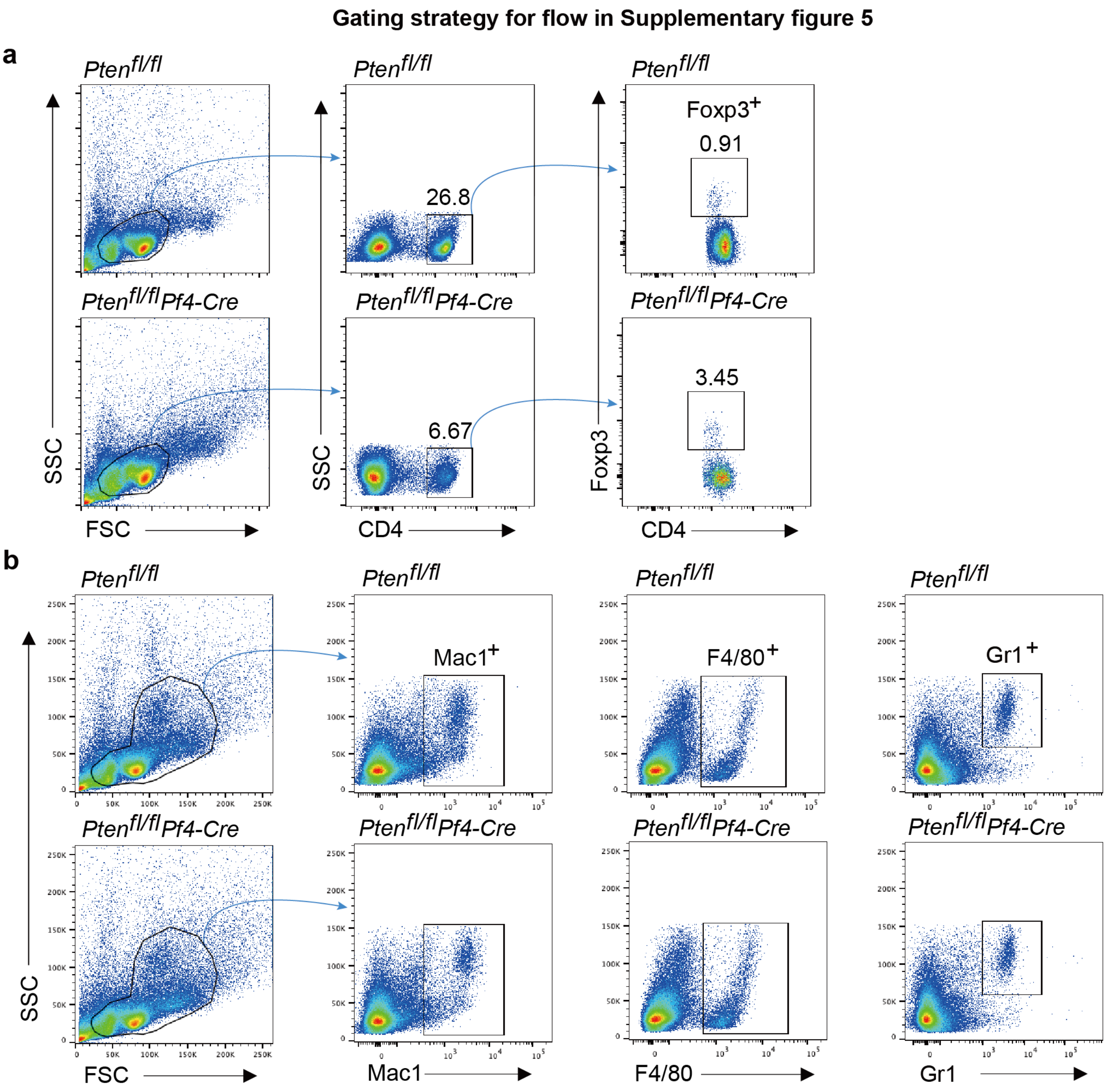

Supplementary Figure. 10

Supplementary Figure 10 Gating Strategy used for flow cytometric analysis in the Supplementary figure 5. All cell populations were gated on live singlets. **a,** The Foxp3^{+} gating panels correspond to FACS data panels in Supplementary figure 5 the third row. **b** The Mac1^{+}, F4/80^{+} or Gr1^{+} gating panels correspond to FACS data panels in the 4th-6th rows of Supplementary figure 5, respectively.

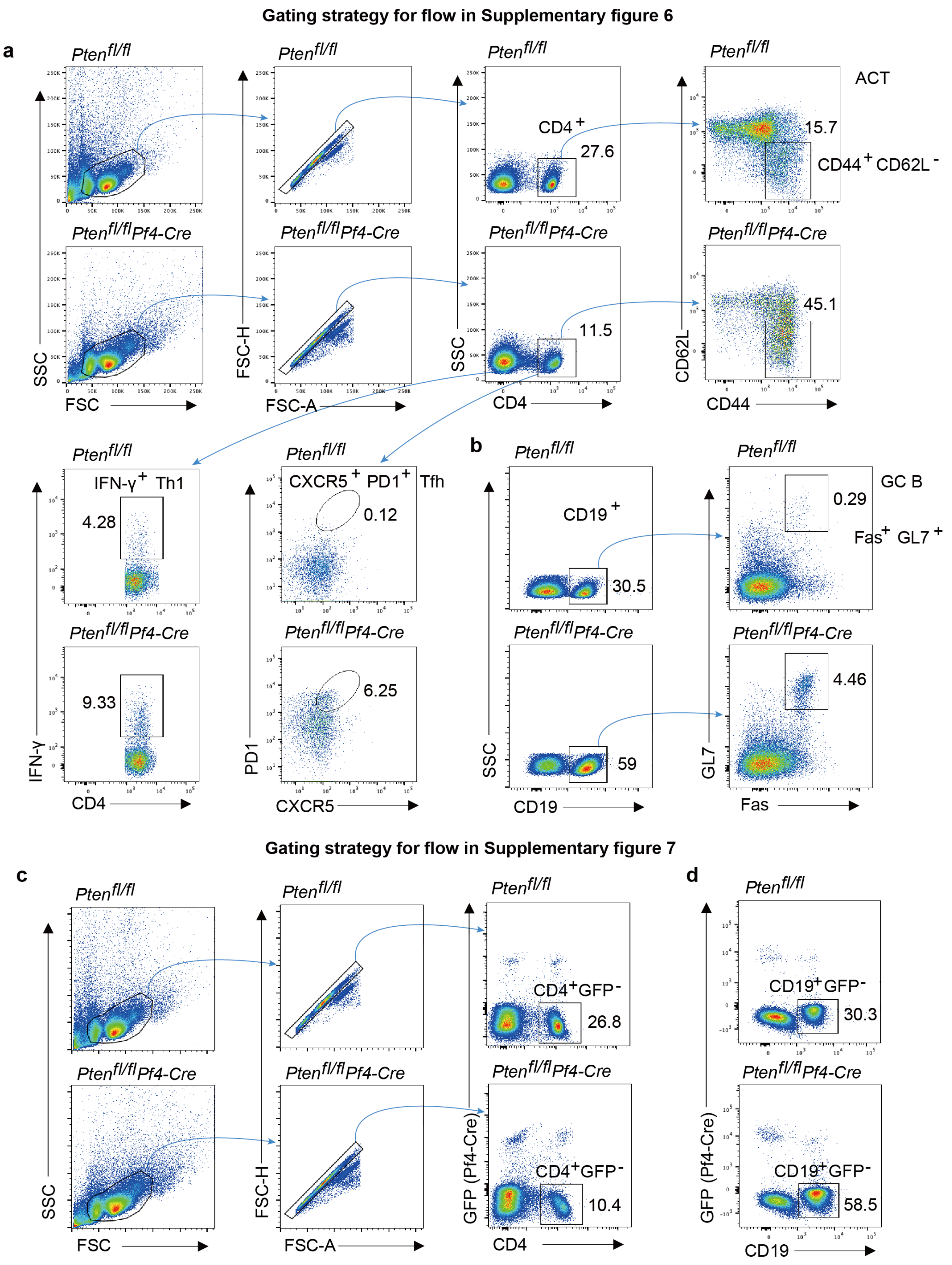

Supplementary Figure. 11

Supplementary Figure 11 Gating Strategy use for flow cytometric analysis in Supplementary figure 6 and 7. All cell populations were gated on live singlets. **a** The CD4^{+} CD44^{+}CD62L^{-} active T cell (ACT), CD4^{+} IFN-γ^{+} Th1, and CD4^{+} CXCR5^{+}PD1^{+} Tfh gating panels correspond to FACS data panels in the Supplementary figure 6a, b, respectively. **b** The CD19^{+} Fas^{+}GL7^{+} Germinal Center B cells (GC B) gating panels correspond to FACS data panels in the Supplementary figure 6c. **c** The CD4^{+}GFP^{-} gating panels correspond to FACS data panels in the Supplementary figure 7a, 7b. **d** The CD19^{+}GFP^{-} gating panels correspond to FACS data panels in the Supplementary figure 7c.

Supplementary Table 1:

REAGENT or ANTIBODY	SOURCE	IDENTIFIER<br>Catlog (Cat)	Dilution
**Antibodies**			
(FITC) anti-B220	(BD Biosciences)	Cat: 553088	1:100
allophycocyanin (APC) anti-IgM	(BD Biosciences)	Cat: 550676	1:100
phycoerythrin (PE) anti-CD24	(BD Biosciences)	Cat: 553262	1:100
FITC anti-CD41	(BD Biosciences)	Cat: 553848	1:100
Percp-Cy5.5 anti-BCL6	(BD Biosciences)	Cat: 562198	1:100
PE-Cy7 anti-CXCR5	(BD Biosciences)	Cat: 560617	1:100
APC anti-IL4	(BD Biosciences)	Cat: 554436	1:200
APC-Cy7 anti-IL17a	(BD Biosciences)	Cat: 560821	1:100
BV421 anti-IFN-γ	(BD Biosciences)	Cat: 563376	1:100
FITC anti P-selectin	(BD Biosciences)	Cat: 553744	1:100
Pacific Blue anti-CD4	(Biolegend)	Cat:100428	1:100
Percp-Cy5.5 anti-CD19	(Biolegend)	Cat: 115534	1:100
APC anti-CXCR5	(Biolegend)	Cat:145506	1:100
APC anti-CD44	(Biolegend)	Cat: 103011	1:200
APC-Cy7 anti-CD62L	(Biolegend)	Cat: 104427	1:100
PE-Cy7 anti-CD43	(Biolegend)	Cat: 121218	1:1000
Pacific Blue anti-GL7	(Biolegend)	Cat:144613	1:200
APC anti-Fas/CD95	(eBioscience)	Cat: 17-0951-82	1:100
AF700 anti-Foxp3	(eBioscience)	Cat: 56-5773-82	1:100
APC anti-Cd42d (GPV)	(eBioscience)	Cat: 17-0421-82	1:100
anti-CD16/CD32	(Biolegend)	Cat:101320	1:100
PE CD62L Monoclonal Antibody	(Invitrogen)	Cat: MA5-17803	1:100
APC anti-Mac1	(BD Biosciences)	Cat: BD553312	1:200
PE anti-CD41	(BD Biosciences)	Cat: BD558040	1:100
PE anti-CD138	(BD Biosciences)	Cat: 561070	1:500

PerCP/Cy5.5 anti-mouse F4/80 Antibody	(Biolegend)	Cat: 123128	1:100
APC/Cy7 anti-mouse CD8a Antibody	(Biolegend)	Cat: 100714	1:200
APC anti-mouse CD45 Antibody	(eBioscience/Invitrogen)	Cat: 17-0451-82	1:200
APC CD41 Monoclonal Antibody	(Invitrogen)	Cat: 17-0411-82	1:100
PE-Cy7 Rat Anti-Mouse Gr-1	(BD Biosciences)	Cat: 552985	1:100
Biotin AH anti-mouse CD11c	(eBioscience)	Cat: 13-0114-82	1:100
PerCP-Cy5.5 Streptavidin	(Biolegend)	Cat: 405214	1:200
FITC anti-mouse MHC II	(eBioscience)	Cat: 11-5321-81	1:100
APC Anti-mouse CD86	(eBioscience)	Cat: 17-0862-81	1:100
PE anti-mouse CD80	(eBioscience)	Cat: 12-0801-82	1:100
PE-Cy7 anti-mouse CD11b	(eBioscience)	Cat: 25-0112-81	1:100
anti-AKT-Thr308	(Cell Signaling Technology, CST)	Cat: 9275L	1:1000
anti-AKT-Ser473	(CST)	Cat: 4060	1:1000
anti-SNAP23-Ser95	(GenScript)	Order No.:C7010CG250	1:1000
anti-Sin1-Thr86	(CST)	Cat: 14716s	1:500
anti-SNAP23	(Proteintech)	Cat: 10825-1-AP	1:500
anti-PTEN	(CST)	Cat: 9559L	1:1000
Rabbit anti-GAPDH	(CST)	Cat: 2118s	1:1000
anti-CD62P (P-selectin) antibody	(Abcam)	Cat: ab255822	1:1000
Biotinylated Peanut Agglutinin	(Vector laboratories)	Cat: B-1075	1:200
anti-B220	(Biolegend)	Cat:103202	1:100
Alexa Fluor 647 conjugated Goat anti-Rat IgG	(Life Technologies)	Cat: A21247	1:500

APC Streptavidin	(BD Biosciences)	Cat: 554067	1:500
Purified anti-mouse CD4 Antibody	(Biolegend)	Cat:100401	1:200
anti-CD31	(BD Biosciences)	Cat: 550274	1:100
Mouse LYVE-1 Biotinylated Antibody	(R&D Systems)	Cat: BAF2125	1:100
PE conjugated Goat anti-rat IgG	(Rockland)	Cat: 612-108-120	1:250
Goat anti-rabbit IgG (H+L) HRP conjugated	(Invitrogen)	Cat: A16110	1:10000
AP-conjugated anti-mouse IgG	(Jackson ImmunoResearch)	Cat: 115-055-146	1:500
anti-β-Actin antibody	(Bioworld)	Cat: AP0060	1:10000
**Commercial Reagents**			
Apyrase	(Sigma)	Cat: A6535-2KU	
PGE1	(Sigma)	Cat: P5515	
ADP	(Ameresco)	Cat: 0160	
human alpha Thrombin	(Enzyme research south bend in USA)	Cat: HT 4082A	
Collagen	(Chrono Log CHRONO-PAR®)	Cat: NC9533954	
PDK1 Inhibitor II	(Calbiochem)	Cat: 521276	
PP242 hydrate	(Sigma)	Cat: P0037	
AKT Inhibitor III (SH6)	(Merck)	Cat: 124009	
ProLong Gold antifade reagent-special packaging	(Invitrogen)	Cat: P36934	
Protease Inhibitor Cocktail	(Roche Life Science)	Cat: 04 693 132 001	
Phosphatase Inhibitor Cocktail	(Roche Life Science)	Cat: 04 096 837 001	
anti-CD3 functional grade	(Invitrogen)	Cat: 16-0031-85	
anti-CD28 functional grade	(Invitrogen)	Cat: 16-0281-85	

Source data for the blot in Supplementary Figure 8b

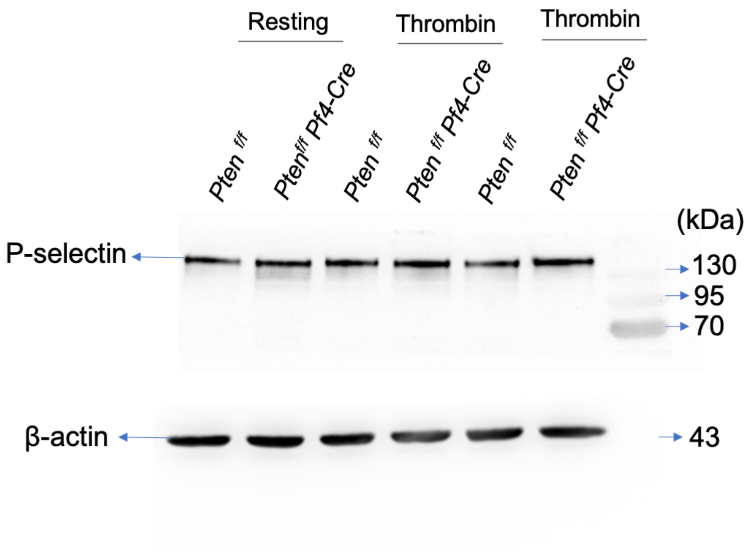

Supplement: Supplementary file 1 — Supplementary Information [file 41467_2022_30444_MOESM1_ESM.pdf]
