# Supplementary material for: The phosphatase PTEN links platelets with immune regulatory functions of mouse T follicular helper cells
Source: Nat Commun. 2022 May 19;13:2762. doi: 10.1038/s41467-022-30444-y (PMC9120038; doi:10.1038/s41467-022-30444-y)
Supplement: Supplementary file 3 — Reporting Summary [file 41467_2022_30444_MOESM3_ESM.pdf]

nature research

Corresponding author(s): Junling Liu, Chuanxin Huang, Xue Chen

Last updated by author(s): Mar 8, 2022

Reporting Summary

Nature Research wishes to improve the reproducibility of the work that we publish. This form provides structure for consistency and transparency in reporting. For further information on Nature Research policies, see our Editorial Policies and the Editorial Policy Checklist.

Statistics

For all statistical analyses, confirm that the following items are present in the figure legend, table legend, main text, or Methods section.

n/a	Confirmed	
☐	☒	The exact sample size (n) for each experimental group/condition, given as a discrete number and unit of measurement
☐	☒	A statement on whether measurements were taken from distinct samples or whether the same sample was measured repeatedly
☐	☒	The statistical test(s) used AND whether they are one- or two-sided<br>*Only common tests should be described solely by name; describe more complex techniques in the Methods section.*
☒	☐	A description of all covariates tested
☐	☒	A description of any assumptions or corrections, such as tests of normality and adjustment for multiple comparisons
☐	☒	A full description of the statistical parameters including central tendency (e.g. means) or other basic estimates (e.g. regression coefficient) AND variation (e.g. standard deviation) or associated estimates of uncertainty (e.g. confidence intervals)
☒	☐	For null hypothesis testing, the test statistic (e.g. F, t, r) with confidence intervals, effect sizes, degrees of freedom and P value noted<br>*Give P values as exact values whenever suitable.*
☒	☐	For Bayesian analysis, information on the choice of priors and Markov chain Monte Carlo settings
☒	☐	For hierarchical and complex designs, identification of the appropriate level for tests and full reporting of outcomes
☒	☐	Estimates of effect sizes (e.g. Cohen's d, Pearson's r), indicating how they were calculated

Our web collection on statistics for biologists contains articles on many of the points above.

Software and code

Policy information about availability of computer code

Data collection	Flow cytometry data was collected on LSR Fortessa (BD Biosciences);<br>Leica TCS Sp8 STED and Leica LAS X Core (3.7.2.22383) software for confocal fluorescence microscopy data collection;<br>HITACHI H-7650 or PHILIPS CM-120 and RADIUS, 2.0 (EMSIS GmbH, Muenster, Germany) software for transmission electron microscope data collection;<br>RNA-seq: platform BGISEQ-500 sequencer;<br>RSEM v1.2.30 (https://github.com/deweylab/RSEM/releases);<br>GSEA v4.2.3 (http://www.gsea-msigdb.org/gsea/downloads.jsp);<br>Bowtie2 v 2.3.4.2 (http://bowtie-bio.sourceforge.net/bowtie2/index.shtml);<br>IPA (according to IPA Ingenuity Web Site, www.ingenuity.com. Ingenuity Systems Inc., Redwood City, CA)<br>edgeR (v3.20.9) (Robinson et al., 2010)<br>Limma v3.42.2<br>cluster Profiler v3.14.3
Data analysis	Flow cytometry data was analyzed using FlowJo (v10) software.<br>Statistical analysis and graphs were generated using GraphPad Prism v7.<br>IF staining images and western blot image quantitation were performed by ImageJ (1.52a).<br>RNA-seq analysis was followed the software documentations, no special changes.

For manuscripts utilizing custom algorithms or software that are central to the research but not yet described in published literature, software must be made available to editors and reviewers. We strongly encourage code deposition in a community repository (e.g. GitHub). See the Nature Research guidelines for submitting code & software for further information.

Data

Policy information about availability of data

All manuscripts must include a data availability statement. This statement should provide the following information, where applicable:

- Accession codes, unique identifiers, or web links for publicly available datasets
- A list of figures that have associated raw data
- A description of any restrictions on data availability

The RNA-seq data have been deposited at the Gene Expression Omnibus (GEO accession number: GSE143698). The gene signature up-regulated in Tfh cells relative to their expression in non-Tfh cells were from GEO (GEO accession-code: GSE21379).
mouse genome (mm9) (ftp://hgdownload.cse.ucsc.edu/goldenPath/mm9).
All the data of this study are available.

Field-specific reporting

Please select the one below that is the best fit for your research. If you are not sure, read the appropriate sections before making your selection.

[x] Life sciences [ ] Behavioural & social sciences [ ] Ecological, evolutionary & environmental sciences

For a reference copy of the document with all sections, see nature.com/documents/nr-reporting-summary-flat.pdf

Life sciences study design

All studies must disclose on these points even when the disclosure is negative.

Sample size	Sample size (or number of repeats) was chosen based on what is common in the field, and what was practical to do. Sample size was determined to be adequate based on the reproducibility between independent experiments. If differences between groups were closed to statistical significance (P = 0.1-0.05), more mice were used for a more rigorous test of statistical analysis.
Data exclusions	No data were excluded from the analyses.
Replication	All experiments were replicated independently and all experiments were reliably reproduced. Number of repeats is provided in the text and figure legends where appropriate.
Randomization	age- and sex-matched animals were used in all experiments.
Blinding	The investigators were blinded during data collection and/or analysis.

Reporting for specific materials, systems and methods

We require information from authors about some types of materials, experimental systems and methods used in many studies. Here, indicate whether each material, system or method listed is relevant to your study. If you are not sure if a list item applies to your research, read the appropriate section before selecting a response.

Materials & experimental systems

n/a	Involved in the study	
[ ]	[x]	Antibodies
[x]	[ ]	Eukaryotic cell lines
[x]	[ ]	Palaeontology and archaeology
[ ]	[x]	Animals and other organisms
[x]	[ ]	Human research participants
[x]	[ ]	Clinical data
[x]	[ ]	Dual use research of concern

Methods

n/a	Involved in the study	
[x]	[ ]	ChIP-seq
[ ]	[x]	Flow cytometry
[x]	[ ]	MRI-based neuroimaging

Antibodies

Antibodies used

Application: Flow cytometry (FC) - Quality routinely tested by the manufactory

1. (FITC) anti-B220 (BD Biosciences) Cat: 553088, clone: RA3-6B2, lot: 5064828, Species: Rat, Application: FC (Routinely Tested), dilution: 1:100
2. allophycocyanin (APC) anti-IgM (BD Biosciences) Cat: 550676, Clone: II/41 (RUO), lot: 5159597, Species: Rat, Application: Flow cytometry (Routinely Tested), dilution: 1:100
3. phycoerythrin (PE) anti-CD24, (BD Biosciences) Cat: 553262, clone: M1/69 (RUO), lot:84497, Species: Rat, Application: Flow

cytometry (Routinely Tested), dilution: 1:100

4. FITC anti-CD41, (BD Biosciences) Cat: 553848, clone: MWReg30, lot:17652, Species: Rat, Application: Flow cytometry (Routinely Tested) , dilution: 1:100
5. Percp-Cy5.5 anti-BCL6, (BD Biosciences) Cat: 562198, clone: K112-91, lot: 6070699, Species: Mouse, Application: Flow cytometry (Routinely Tested) , dilution: 1:100
6. PE-Cy7 anti-CXCR5, (BD Biosciences) Cat: 560617, clone:2G8, lot:5071937, Species: Mouse, Application: Flow cytometry (Routinely Tested), dilution: 1:100
7. APC anti-IL4, (BD Biosciences) Cat: 554436, clone: 11B11, lot:6070957, Species: Rat, Application: Flow cytometry (Routinely Tested) (dilution 1:200)
8. APC-Cy7 anti-IL17a, (BD Biosciences) Cat: 560821, clone:TC11-18H10, lot:5191521, Species: Rat, Application: Flow cytometry (Routinely Tested), dilution: 1:100
9. BV421 anti-IFN-γ, (BD Biosciences) Cat: 563376, clone: XMG1.2, Species: Rat, Application: Flow cytometry (Routinely Tested), dilution: 1:100
10. FITC anti P-selectin, (BD Biosciences) Cat: 553744, clone: RB40.34 lot:7188677, Species: Rat, Application: Flow cytometry (Routinely Tested), dilution: 1:100
11. Pacific Blue anti-CD4, (Biolegend) Cat:100428, clone: GK1.5, lot: B212372, Species: Rat, Application: Flow cytometry (Routinely Tested), dilution: 1:100
12. Percp-Cy5.5 anti-CD19, (Biolegend) Cat: 115534, clone:6D5, lot: B171376, Species: Rat, Application: Flow cytometry (Routinely Tested) dilution: 1:100
13. APC anti-CXCR5, (Biolegend) Cat:145506, clone: L138D7, lot: B215836, Species: Rat, Application: Flow cytometry (Routinely Tested) dilution: 1:100
14. APC anti-CD44, (Biolegend) Cat: 103011, clone: IM7, lot: B207776, Species: Rat, Application: Flow cytometry (Routinely Tested), dilution 1:200
15. APC-Cy7 anti-CD62L, (Biolegend) Cat: 104427, clone: MEL-14, lot: B229840, Species: Rat, Application: Flow cytometry (Routinely Tested) dilution: 1:100
16. PE-Cy7 anti-CD43, (Biolegend) Cat: 121218, clone: 1B11, lot: B164693, Species: Rat, Application: Flow cytometry (Routinely Tested), dilution 1:1000
17. Pacific Blue anti-GL7, (Biolegend) Cat:144613, clone:GL7, lot: B209952, Species: Rat, Application: Flow cytometry (Routinely Tested), dilution 1:200
18. APC anti-Fas/CD95, (eBioscience) Cat: 17-0951-82, clone:15A7, lot: E13624-109, Species: Mouse, Application: Flow cytometry. dilution: 1:100
19. AF700 anti-Foxp3, (eBioscience) Cat: 56-5773-82 , clone: FJK-16s, lot: E09023-1634, Species: Rat, Application: Flow Cytometry, Immunocytochemistry (ICC), Immunofluorescence (IF), Immunohistochemistry (IHC), Immunohistochemistry (Paraffin) (IHC (P)), Immunoprecipitation (IP) dilution: 1:100
20. APC anti-Cd42d (GPV) (eBioscience) Cat: 17-0421-82, clone: 1C2, lot: Species: Armenian Hamster, Application: Flow cytometry. dilution: 1:100
21. anti-CD16/CD32 (Biolegend) Cat:101320, clone:93, lot: B164564, Species: Rat, Application: Flow cytometry (Routinely Tested) dilution: 1:100
22. PE CD62L Monoclonal Antibody (Invitrogen), Clone: MEL-14, Cat: MA5-17803, monoclonal antibody, Lot: E013141632, Species: Rat, Application: Flow cytometry (Routinely Tested) dilution: 1:100
23. APC anti-Mac1(BD Biosciences): clone M1/70, Cat: BD553312, Reactivity: Mouse (QC Testing), Human (Tested in Development), Species: Rat, Application: Flow cytometry (Routinely Tested), dilution 1:200
24. PE anti-CD41(BD Biosciences), Clone: MWReg30, Cat: BD558040, monoclonal antibody, Species: Rat, Application: Flow cytometry (Routinely Tested) dilution: 1:100
25. PE anti-CD138 (BD Biosciences): clone 281-2, Cat: 561070, Lot: 9023581, Species: Rat, Application: Flow cytometry (Routinely Tested) dilution 1:500
26. PerCP/Cy5.5 anti-mouse F4/80 Antibody (Biolegend), Clone BM8, Cat: 123128, monoclonal antibody, Species: Rat, Application: Flow cytometry (Routinely Tested) dilution: 1:100
27. APC/Cy7 anti-mouse CD8a Antibody (Biolegend), Clone: 53-6.7, Cat: 100714, monoclonal antibody, Lot: B162658, Species: Rat, Application: Flow cytometry (Routinely Tested) dilution 1:200
28. APC anti-mouse CD45 Antibody (eBioscience/Invitrogen), Clone: 30-F11, Cat: 17-0451-82, monoclonal antibody, Lot: 4291970, Species: Rat, Application: Flow cytometry (Routinely Tested) dilution 1:200
29. APC CD41 Monoclonal Antibody (Invitrogen), Clone: eBioMWReg30, Cat: 17-0411-82, monoclonal antibody, Species: Rat, Application: Flow cytometry (Routinely Tested) dilution: 1:100
30. PE-Cy7 Rat Anti-Mouse Gr-1 (BD Biosciences), Cat: 552985, monoclonal antibody, Species: Rat, Application: Flow Cytometry. dilution: 1:100
31. Biotin AH anti-mouse CD11c (eBioscience), Clone: N418, Cat: 13-0114-82, Lot: 4292345, Species: AH IgG, Application: Flow cytometry (Routinely Tested). dilution: 1:100
32. PerCP-Cy5.5 Streptavidin (Biolegend), Cat: 405214, Lot: B217949. dilution: 1:200
33. FITC anti-mouse MHC II (I-A/I-E) (eBioscience), Clone: M5/114.15.2, Cat: 11-5321-81, Lot: 4273292, Species: Rat IgG2b, Application: FC (Routinely Tested). dilution: 1:100
34. APC Anti-mouse CD86 (eBioscience), Clone: GL1, Cat: 17-0862-81, Lot: 7082942, Species: Rat IgG2a, kappa, Application: Flow cytometry (Routinely Tested). dilution: 1:100
35. PE anti-mouse CD80 (eBioscience), Clone: 16-10A1, Cat: 12-0801-82, Lot: 4289574, Species: Armenian hamster/IgG, Application: Flow cytometry (Routinely Tested). dilution: 1:100
36. PE-Cy7 anti-mouse CD11b (eBioscience), Clone: M1/70, Cat: 25-0112-81, Lot: 4285186, Species: Rat / IgG2b, kappa, Application: Flow cytometry (Routinely Tested). dilution: 1:100

Application: WB - Quality tested by the manufactory

37. anti-AKT-Thr308 (Cell Signaling Technology, CST), Cat: 9275L, lot: 3, Species: Rabbit, Application: Immunoprecipitation, Western (dilution 1:1000)

38. anti-AKT-Ser473 (CST), Cat: 4060, lot: 6, Species: Rabbit, Application: Flow Cytometry, Immunofluorescence, Immunohistochemistry, immunoprecipitation, Western (dilution 1:1000)
39. anti-SNAP23-Ser95 (GenScript), Peptide Order No.:C7010CG250, Species: Rabbit, Application: WB Quality tested (dilution 1:1000)
40. anti-Sin1-Thr86 (CST), Cat: 14716s, Species: Rabbit, Application: immune-precipitation, Western (dilution 1:500)
41. anti-SNAP23 (Proteintech), Cat: 10825-1-AP, Species: Rabbit, Applications: WB, IP, IHC, IF, FC, ELISA (dilution 1:500)
42. anti-PTEN (CST), Cat:9559L, lot:7, Species: Rabbit, Application:
Immunohistochemistry, Immunoprecipitation, Western (dilution 1:1000)
43.Rabbit anti-GAPDH (CST), Cat:2118s, clone: 14C10, lot: Species: Rabbit, Application: Flow Cytometry, Immunofluorescence, Immunohistochemistry, Western (dilution 1:1000)
44. anti-CD62P (P-selectin) antibody (Abcam), Clone: EPR22850-190, Cat: ab255822, monoclonal antibody, Lot: GR3383644-1, Species: Rabbit, Application: WB, IHC-P. (dilution 1:1000)

Application: IHC/IF - Quality tested by the manufactory

45. Biotinylated Peanut Agglutinin (PNA, Vector laboratories) Cat: B-1075, lot: X1221, Application: Immunohistochemistry/ Immunocytochemistry, Immunofluorescence, Blotting Applications, Elispot, ELISAs, Glycobiology. dilution 1:200
46. anti-B220 (Biolegend), Cat:103202, Clone: RA3-6B2, lot: B123193, Species: Rat, Application: FC-Quality tested CyTOF®; IHC-F–Verified. dilution 1:100.
47. Alexa Fluor 647 conjugated Goat anti-Rat IgG (Life Technologies) Cat: A21247, Polyclonal, lot: 1654305, Species: Goat, Application: IF, IHC, Flow. dilution 1:500
48. APC Streptavidin (BD Biosciences) Cat: 554067. dilution 1:500
49. Purified anti-mouse CD4 Antibody (Biolegend), Clone: GK1.5, Cat:100401, monoclonal antibody, Species: Rat. dilution 1:200
50. anti-CD31 (BD Biosciences): Clone MEC13.3, Cat: 550274, Species: Rat, Application: Flow cytometry (Routinely Tested), Immunohistochemistry-frozen, dilution 1:100
51. Mouse LYVE-1 Biotinylated Antibody (R&D Systems), Cat: BAF2125, Polyclonal Goat IgG, Species: Goat, Application: WB, IHC. dilution 1:100
52. PE conjugated Goat anti-rat IgG (Rockland) Cat: 612-108-120. Dilution 1:250
53. Goat anti-rabbit IgG (H+L) Highly Cross-Adsorbed Secondary antibody HRP conjugated (Invitrogen) Cat: A16110, Dilution 1:10000
54. AP-conjugated anti-mouse IgG (Jackson ImmunoResearch), Cat: 115-055-146, Dilution 1:500
55. anti-CD3 functional grade antibody (Invitrogen), Catlog: 16-0031-85
56. anti-CD28 functional grade antibody (Invitrogen), Catlog: 16-0281-85
57. anti-β-Actin antibody (Bioworld), Catlog: AP0060 1:10000

Validation

Application: Flow cytometry (FC) - Quality routinely tested by the supplier

1. (FITC) anti-B220 (BD Biosciences) Cat: 553088 https://www.citeab.com/antibodies/2411763-553088-bd-pharmingen-fitc-rat-anti-mouse-cd45r-b220
2. allophycocyanin (APC) anti-IgM (BD Biosciences) Cat: 550676 https://www.citeab.com/antibodies/2411763-553088-bd-pharmingen-fitc-rat-anti-mouse-cd45r-b220
3. phycoerythrin (PE) anti-CD24, (BD Biosciences) Cat: 553262 (RUO)
https://www.citeab.com/antibodies/2410070-553262-bd-pharmingen-pe-rat-anti-mouse-cd24?des=32e2e2082028c5e3
4. FITC anti-CD41,（BD Biosciences） Cat: 553848
https://www.citeab.com/antibodies/2410014-553848-bd-pharmingen-fitc-rat-anti-mouse-cd41?des=98a92d0e27e527f6
5. Percp-Cy5.5 anti-BCL6, (BD Biosciences) Cat: 562198
https://www.citeab.com/antibodies/2409220-562198-bd-pharmingen-percp-cy-5-5-mouse-anti-bcl-6?des=d666bd21339e5be8
6. PE-Cy7 anti-CXCR5, (BD Biosciences) Cat: 560617
https://www.citeab.com/antibodies/2411096-560617-bd-pharmingen-pe-cy-7-rat-anti-mouse-cd185?des=6f00068c58a460d0
7. APC anti-IL4, (BD Biosciences) Cat: 554436
https://www.citeab.com/antibodies/2413710-554436-bd-pharmingen-apc-rat-anti-mouse-il-4?des=ba3c80da83aa6e69
8. APC-Cy7 anti-IL17a, (BD Biosciences) Cat: 560821
https://www.citeab.com/antibodies/2406876-560821-bd-pharmingen-apc-cy-7-rat-anti-mouse-il-17a?des=68fdc3a0f2760d81
9. BV421 anti-IFN-γ, (BD Biosciences) Cat: 563376
https://www.citeab.com/antibodies/2409490-563376-bd-horizon-bv421-rat-anti-mouse-ifn?des=4b432b162b8f12d8
10. FITC anti P-selectin, (BD Biosciences) Cat: 553744
https://www.citeab.com/antibodies/2410220-553744-bd-pharmingen-fitc-rat-anti-mouse-cd62p?des=816645ee5bf47e22
11. Pacific Blue anti-CD4, (Biolegend) Cat:100428
https://www.citeab.com/antibodies/517127-100428-pacific-blue-anti-mouse-cd4-antibody?des=dc184b2dcf85680d
12. Percp-Cy5.5 anti-CD19, (Biolegend) Cat: 115534
https://www.citeab.com/antibodies/518004-115534-percp-cyanine5-5-anti-mouse-cd19-antibody?des=818194cd4dafeef7
13. APC anti-CXCR5, (Biolegend) Cat:145506
https://www.citeab.com/antibodies/1483674-145506-apc-anti-mouse-cd185-cxcr5-antibody?des=dcc5fda300936ded
14. APC anti-CD44, (Biolegend) Cat: 103011
https://www.citeab.com/antibodies/517918-103011-apc-anti-mouse-human-cd44-antibody?des=14ad2fecd96f9180
15. APC-Cy7 anti-CD62L, (Biolegend) Cat: 104427
https://www.citeab.com/antibodies/518392-104427-apc-cyanine7-anti-mouse-cd62l-antibody?des=8ac53641fa04cee7
16. PE-Cy7 anti-CD43, (Biolegend) Cat: 121218
https://www.citeab.com/antibodies/518737-121218-pe-cyanine7-anti-mouse-cd43-activation-associa?des=4159c0e440ad4ffa
17. Pacific Blue anti-GL7, (Biolegend) Cat:144613
https://www.citeab.com/antibodies/2083023-144613-pacific-blue-anti-mouse-human-gl7-antigen-t?des=1ee3defeaea5d901
18. APC anti-Fas/CD95, (eBioscience) Cat: 17-0951-82

https://www.citeab.com/antibodies/2039453-17-0951-82-cd95-apo-1-fas-monoclonal-antibody-15a?des=5a4f184b36c6f2fe
19. AF700 anti-Foxp3, (eBioscience) Cat: 56-5773-82
https://www.citeab.com/antibodies/2041735-56-5773-82-foxp3-monoclonal-antibody-fjk-16s-alex?des=f0719ecd65a98ed8
16. APC anti-Cd42d (GPV) (eBioscience) Cat: 17-0421-82
https://www.citeab.com/antibodies/2038769-17-0421-82-cd42d-monoclonal-antibody-1c2-apc-ebi?des=ac72ec51accdc58d
21. anti-CD16/CD32 (Biolegend) Cat:101320
https://www.citeab.com/antibodies/517402-101320-trustain-fcx-anti-mouse-cd16-32-antibody?des=7c26517276959644
22. PE CD62L Monoclonal Antibody (Invitrogen), Cat: MA5-17803
https://www.citeab.com/antibodies/1914201-ma5-17803-cd62l-monoclonal-antibody-mel-14-pe?des=4bd5c36ea9430e6e
23. APC anti-Mac1(BD Biosciences): clone M1/70, Cat: 553312
https://www.citeab.com/antibodies/2408215-553312-bd-pharmingen-apc-rat-anti-cd11b?des=93cc718e1438f50b
23. PE anti-CD41(BD Biosciences), Cat: 558040
https://www.citeab.com/antibodies/2410962-558040-bd-pharmingen-pe-rat-anti-mouse-cd41?des=baa4c50b130eaab4
25. PE anti-CD138 (BD Biosciences): Cat: 561070
https://www.citeab.com/antibodies/2408745-561070-bd-pharmingen-pe-rat-anti-mouse-cd138?des=239232987dbbd816
26. PerCP/Cy5.5 anti-mouse F4/80 Antibody (Biolegend), Cat: 123128
https://www.citeab.com/antibodies/519062-123128-percp-cyanine5-5-anti-mouse-f4-80-antibody?des=853130bbb8558a20
27. APC/Cy7 anti-mouse CD8a Antibody (Biolegend), Cat: 100714
https://www.citeab.com/antibodies/517216-100714-apc-cyanine7-anti-mouse-cd8a-antibody?des=99660e2bfbfa2054
28. APC anti-mouse CD45 Antibody (Invitrogen), Cat: 17-0451-82
https://www.citeab.com/antibodies/2038853-17-0451-82-cd45-monoclonal-antibody-30-f11-apc-e?des=fb9796b2f857d4c5
29. APC CD41 Monoclonal Antibody (Invitrogen), Cat: 17-0411-82
https://www.citeab.com/antibodies/2038692-17-0411-82-cd41a-monoclonal-antibody-ebiomwreg30-m?des=c52e2c775c38043d
30. PE-Cy7 Rat Anti-Mouse Gr-1 (BD Biosciences) Cat: 552985
https://www.citeab.com/antibodies/2411828-552985-bd-pharmingen-pe-cy-7-rat-anti-mouse-ly-6g-a?des=4f43c29b02a5081a
31. Biotin AH anti-mouse CD11c (eBioscience), Cat: 13-0114-82
https://www.citeab.com/antibodies/2038168-13-0114-82-cd11c-monoclonal-antibody-n418-biotin?des=4e7df1637e759939
32. PerCP-Cy5.5 Streptavidin (Biolegend), Cat: 405214
https://www.biolegend.com/en-us/products/percp-cyanine5-5-streptavidin-4212
33. FITC anti-mouse MHC II (I-A/I-E) (eBioscience), Cat: 11-5321-81
https://www.thermofisher.com/antibody/product/MHC-Class-II-I-A-I-E-Antibody-clone-M5-114-15-2-Monoclonal/11-5321-81
34. APC Anti-mouse CD86 (eBioscience), Clone: GL1, Cat: 17-0862-81, Lot: 7082942, Species: Rat IgG2a, kappa, Application: Flow cytometry (Routinely Tested).
https://www.citeab.com/antibodies/2039360-17-0862-82-cd86-b7-2-monoclonal-antibody-gl1-ap?des=e46ae5b220a535ac
35. PE anti-mouse CD80 (eBioscience), Cat: 12-0801-82
https://www.citeab.com/antibodies/2039297-12-0801-82-cd80-b7-1-monoclonal-antibody-16-10a1?des=652ad622c034e309
36. PE-Cy7 anti-mouse CD11b (eBioscience), Cat: 25-0112-81
https://www.citeab.com/antibodies/2038111-25-0112-82-cd11b-monoclonal-antibody-m1-70-pe-cya?des=bcbec43bad1548a7

Application: WB - Quality tested by the supplier

37. anti-AKT-Thr308 (Cell Signaling Technology, CST), Cat: 9275L
https://www.citeab.com/antibodies/125976-9275-phospho-akt-thr308-antibody?des=436867e9cb87bdab
38. anti-AKT-Ser473 (CST), Cat: 4060
https://www.citeab.com/antibodies/123848-4060-phospho-akt-ser473-d9e-xp-rabbit-mab?des=3cb714006e1b6cb0
39. anti-SNAP23-Ser95 (GenScript), Peptide Order No.:C7010CG250
antibody@genscript.com.cn
40. anti-Sin1-Thr86 (CST), Cat: 14716s
https://www.citeab.com/antibodies/2444925-14716-phospho-sin1-thr86-d4u9l-rabbit-mab?des=85451e6bdc5ba061
41. anti-SNAP23 (proteintech), Cat: 10825-1-AP
https://www.citeab.com/antibodies/976162-10825-1-ap-snap23-antibody?des=1409da2a5c02682d
42. anti-PTEN (CST), Cat:9559L
https://www.citeab.com/antibodies/126240-9559-pten-138g6-rabbit-mab?des=629c7fc7d811db12
43. Rabbit anti-GAPDH (CST), Cat:2118s
https://www.citeab.com/antibodies/122875-2118-gapdh-14c10-rabbit-mab?des=9f20dfa5bc894376
44. anti-CD62P (P-selectin) antibody (Abcam), Cat: ab255822
https://www.citeab.com/antibodies/7431098-ab255822-anti-cd62p-antibody-epr22850-190?des=ea3a7e80582b0d94

Application: IHC/IF - Quality tested by the manufactory

45. biotinylated Peanut Agglutinin (PNA, Vector laboratories) Cat: B-1075
https://vectorlabs.com/products/glycobiology/biotinylated-peanut-agglutinin-pna
46. anti-B220 (Biolegend), Cat:103202
https://www.citeab.com/antibodies/518048-103202-purified-anti-mouse-human-cd45r-b220-antibody?des=0fe160d0ea483d60
47. Alexa Fluor 647 conjugated Goat anti-Rat IgG (Life Technologies) Cat: A-21247,
https://www.citeab.com/antibodies/2401279-a-21247-goat-anti-rat-igg-h-l-cross-adsorbed-secon?des=6a2ffd6dccf71d1b
48. APC Streptavidin (BD Biosciences) Cat: 554067.
https://www.bdbiosciences.com/en-au/products/reagents/flow-cytometry-reagents/research-reagents/single-color-antibodies-ruo/apc-streptavidin.554067

49. Purified anti-mouse CD4 Antibody (Biolegend), Cat:100401
https://www.citeab.com/antibodies/517103-100401-purified-anti-mouse-cd4-antibody?des=ce1c672fc0db9961
50. anti-CD31 (BD Biosciences), Cat: 550274
https://www.citeab.com/antibodies/3288830-550274-bd-pharmingen-purified-rat-anti-mouse-cd31?des=8c949d1dc6222b5f
51. Mouse LYVE-1 Biotinylated Antibody (R&D Systems), Cat: BAF2125
https://www.citeab.com/antibodies/691816-baf2125-mouse-lyve-1-biotinylated-antibody?des=20e529a4d3ea864a
52. PE conjugated Goat anti-rat IgG (Rockland) Cat: 612-108-120. Dilution 1:250
https://www.clinisciences.com/en/other-products-186/anti-rat-igg-h-l-goat-antibody-528009664.html
53. Goat anti-rabbit IgG (H+L) Highly Cross-Adsorbed Secondary antibody HRP conjugated (Invitrogen) Cat: A16110, Dilution 1:10000
https://www.thermofisher.com/antibody/product/Goat-anti-Rabbit-IgG-H-L-Highly-Cross-Adsorbed-Secondary-Antibody-Polyclonal/A16110
54. AP-conjugated anti-mouse IgG (Jackson ImmunoResearch), Cat: 115-055-146
https://shop.lucerna-chem.ch/563445/alkaline-phosphatase-affinipure-goat-anti-mouse-igg-h-l-min-x-hu-bov-hrs-rb-sw-sr-prot
55. anti-CD3 antibody (Invitrogen), Catlog: 16-0031-85
https://www.thermofisher.com/antibody/product/CD28-Antibody-clone-37-51-Monoclonal/16-0281-85
56. anti-CD28 antibody (Invitrogen), Catlog: 16-0281-85
https://www.thermofisher.com/antibody/product/CD28-Antibody-clone-37-51-Monoclonal/16-0281-85
57. anti-β-Actin antibody (Bioworld), Catlog: AP0060 1:10000
https://www.citeab.com/antibodies/2206801-ap0060-actin-i102-polyclonal-antibody

Animals and other organisms

Policy information about studies involving animals; ARRIVE guidelines recommended for reporting animal research

Laboratory animals	All mice were crossed on C57BL/6 background.<br>Pf4 transgenic mice and mice with loxP-flanked Pten alleles (Ptenfl/fl) have been previously described. Rosa26mT/mG (Jax007676) mice were from Nanjing Biomedical Research Institute of Nanjing University. The Rosa26mT/mG reporter mice we used contain a loxP-flanked ('floxed') tandem dimer Tomato gene (mT) and STOP cassette followed by the gene encoding GFP (mG) at the ubiquitously membrane-targeted Rosa26 locus. In this lineage tracing by Cre-Loxp system, PF4-Cre mediated excision of the floxed STOP cassette results in descendible and constitutive expression of GFP. According to different experimental purposes, these mice were crossed to generate Ptenfl/flPf4-Cre, Rosa26mT/mGPf4-Cre, Rosa26mT/mGPtenfl/flPf4-Cre, respectively. The development of these lymphoproliferative and autoimmune diseases was monitored by frequent visual examination, flow cytometry and histopathological analyses.<br>All mice were bred and maintained under a 12-h reverse light/dark cycle and specific pathogen free (SPF) conditions.<br>The animal facility was maintained at a temperature of 22 ± 2 °C with 40-70 % humidity. Age- and sex-matched male and female adult mice were used in each independent experiment. The ages of mice were depended on different experimental design and were clearly described in the manuscript or figure legend.
Wild animals	No wild animals were used in this study.
Field-collected samples	No field-collected samples were used in this study.
Ethics oversight	All mice were housed in specific pathogen-free (SPF) facilities and all animal experiments were performed in strict accordance with protocols approved by Institutional Animal care and Use Committee (IACUC) of Shanghai Jiao Tong University School of Medicine

Note that full information on the approval of the study protocol must also be provided in the manuscript.

Flow Cytometry

Plots

Confirm that:

- [x] The axis labels state the marker and fluorochrome used (e.g. CD4-FITC).
- [x] The axis scales are clearly visible. Include numbers along axes only for bottom left plot of group (a 'group' is an analysis of identical markers).
- [x] All plots are contour plots with outliers or pseudocolor plots.
- [x] A numerical value for number of cells or percentage (with statistics) is provided.

Methodology

Sample preparation	Bone marrow cells were flushed from tibia and femurs with sterile FACS buffer (PBS supplemented with 2% FBS and 2mM EDTA). Lymph nodes and spleens were gently grind against the cell strainer by the plunger from a syringe and single-cell suspensions were filtered through fine mesh. Red blood cells were lysed with AKC lysis buffer. Cells were counted and stained with surface antibodies in FACS buffer, followed by incubation for 30 min on ice. For intracellular staining, cells were fixed and permeabilized using the Foxp3 Fix/Perm kit.
Instrument	Samples were collected on LSR Fortessa cytometer (BD Biosciences).

Software	Flow cytometry data was acquired using BD Diva software. Data analysis was performed using FlowJo (Treestar) software (v10).
Cell population abundance	A mimimum of 20000 CD4+ T cells or other indicated cells collected per sample. The purity of the samples within post-sort fractions was above 99% and determined by a LSR Fortessa cytometer (BD Biosciences).
Gating strategy	For flow cytometric analysis all cell populations were gated on live singlets. Lymphocytes were identified by size and granularity in the FSC/SSC scatter. Dead cells and non-singlet events were excluded by 7-amino-actinomycin D staining and the forward scatter area (FSC-A) versus height (FSC-H) characteristics. Further gating depended on experimental design and were clearly described in the manuscript.

☒ Tick this box to confirm that a figure exemplifying the gating strategy is provided in the Supplementary Information.
